# Temporal Dynamics of Inflammatory, Glial, and Metabolic Biomarkers Following Severe Diffuse Traumatic Brain Injury in a Rat Model

**DOI:** 10.3390/biomedicines13123123

**Published:** 2025-12-18

**Authors:** Ozan Başkurt

**Affiliations:** 1 Department of Neurosurgery, Faculty of Medicine, Istanbul Arel University, 34537 Istanbul, Türkiye; ozanbskrt@gmail.com; 2 Department of Neurosurgery, Memorial Bahçelievler Hospital, 34180 Istanbul, Türkiye

**Keywords:** neurotrauma, traumatic brain injury, TNF-α, IL-6, GFAP, insulin, biomarker dynamics, secondary injury, Marmarou model

## Abstract

**Background:** Traumatic brain injury (TBI) initiates a complex sequence of inflammatory, glial, and metabolic events that evolve dynamically and contribute substantially to secondary brain injury. This study aimed to characterize the temporal serum dynamics of tumor necrosis factor-α (TNF-α), interleukin-6 (IL-6), glial fibrillary acidic protein (GFAP), and insulin following severe diffuse TBI in a rat model, with the goal of delineating the coordinated progression of inflammatory, astroglial, and metabolic responses. **Methods:** Severe diffuse TBI was induced in adult male Sprague–Dawley rats using the Marmarou weight-drop model. Animals were randomized into five groups (sham, 1 h, 6 h, 24 h, 72 h; n = 10 per group). Serum TNF-α, IL-6, GFAP, and insulin levels were quantified using ELISA assays. Group differences were assessed using one-way ANOVA with Tukey’s post hoc test or Kruskal–Wallis analysis with Dunn’s correction where appropriate. Results were expressed as mean ± SD. **Results:** TNF-α demonstrated a biphasic pattern, declining at 6 h before peaking significantly at 24 h (*p* < 0.05) and subsequently decreasing at 72 h. IL-6 exhibited mild suppression at 6 h followed by a significant secondary elevation at 24 h (*p* < 0.05), with persistently elevated levels at 72 h. GFAP showed delayed kinetics, decreasing at 6 h but rising progressively to a peak at 24 h, consistent with subacute astroglial activation. Insulin levels declined at 6 h and increased significantly at 24 h and 72 h (*p* < 0.05), indicating evolving metabolic adaptation. Overall, cytokine activity preceded glial and endocrine changes, revealing a sequential inflammatory–glial–metabolic cascade. **Conclusions:** This study delineates the temporal serum profiles of TNF-α, IL-6, GFAP, and insulin after severe diffuse TBI, revealing a coordinated transition from acute inflammation to astroglial activation and metabolic adaptation. These results support the utility of multimodal biomarker panels for phase-specific characterization of secondary injury and identify GFAP and IL-6 as promising subacute markers with translational relevance. The findings should be interpreted as descriptive temporal patterns rather than mechanistic evidence, pending confirmation with complementary molecular analyses.

## 1. Introduction

Traumatic brain injury (TBI) remains a major global health concern and is a leading cause of morbidity and mortality across all age groups [1]. Beyond the initial mechanical insult, a cascade of secondary injury mechanisms—including inflammation, oxidative stress, and metabolic dysregulation—plays a pivotal role in driving neuronal degeneration and shaping long-term neurological outcomes [2,3]. These processes evolve dynamically, with early cytokine release, astroglial activation, and endocrine disturbances marking distinct temporal phases that critically influence the progression of secondary brain injury.

Among the earliest molecular mediators released after cerebral trauma are the pro-inflammatory cytokines tumor necrosis factor-α (TNF-α) and interleukin-6 (IL-6) [2,4,5]. TNF-α acts as a pleiotropic regulator of blood–brain barrier permeability, leukocyte trafficking, and apoptotic signaling, whereas IL-6 contributes to both pro-inflammatory and neuroprotective responses via activation of STAT3- and CREB-dependent pathways [1,6,7]. The temporal balance between these cytokines is essential in determining the transition from acute neuroinflammation to subsequent glial-driven repair.

Astroglial injury additionally forms a central component of the secondary damage cascade in TBI, reflecting its pivotal role in post-traumatic neurodegeneration. Glial fibrillary acidic protein (GFAP), an astrocyte-specific intermediate filament, enters the systemic circulation following astrocytic structural disruption or blood–brain barrier (BBB) compromise [8]. Accumulating experimental and clinical evidence identifies GFAP as a sensitive and clinically relevant biomarker of glial injury severity, with strong prognostic value in both acute and subacute phases of TBI [8,9,10].

In addition to glial pathology, TBI triggers profound neuroendocrine disturbances that alter systemic insulin and glucose regulation [11]. Acute hypothalamic–pituitary axis dysfunction frequently produces transient hypoinsulinemia, which may subsequently normalize or evolve into compensatory hyperinsulinemia as part of metabolic recovery [12,13]. Beyond its peripheral metabolic role, insulin exerts neurotrophic and neuroprotective effects through Akt- and AMPK-dependent pathways, influencing neuronal survival, synaptic maintenance, and energy homeostasis [14,15].

Although TNF-α, IL-6, GFAP, and insulin have each been individually associated with TBI, few studies have characterized their coordinated temporal dynamics within a single, standardized diffuse injury model. Accordingly, the aim of this study was not to introduce novel biomarkers but to generate a high-resolution temporal profile of four clinically relevant markers during the first 72 h after severe diffuse TBI in rats. Despite extensive literature on their individual functions, integrated and time-synchronized data capturing their collective behavior remain limited. By quantifying these biomarkers at defined early post-injury intervals, this study seeks to refine the temporal relationships among acute inflammatory activation, astroglial injury, and metabolic adaptation. Such temporal mapping provides methodological value for preclinical TBI research, where sampling windows, biomarker selection, and experimental timing critically shape the interpretation of downstream molecular analyses.

## 2. Materials and Methods

### 2.1. Animals

A total of 50 adult male Sprague-Dawley rats aged 12–16 weeks (weight: 270–300 g) were included in the study. Animals were housed under standard laboratory conditions with a 12 h light/dark cycle, controlled temperature (22 ± 2 °C), and 50% humidity, with ad libitum access to food and water. All experimental procedures complied with the European Union Directive 2010/63/EU for the care and use of laboratory animals and were approved by the Yeditepe University Faculty of Medicine Animal Ethics Committee (Istanbul, Türkiye).

### 2.2. Experimental Model

For all procedures, anesthesia was induced with 4–5% isoflurane in 70% N_2_O and 30% O_2_ and maintained at 1.5–2%, titrated to abolish the toe-pinch and corneal reflexes. Adequate anesthetic depth was achieved within 3 min, with a total anesthesia duration of approximately 8–10 min per animal. Physiological variables, including rectal temperature, mean arterial pressure, and arterial blood gases (via femoral artery cannulation), were continuously monitored throughout the procedure.

Diffuse TBI was induced using the Marmarou impact-acceleration model [16,17]. A midline scalp incision was made, and a 10 mm stainless steel disk was affixed between bregma and lambda. Severe diffuse injury was produced by releasing a 500 g weight from a height of 2.1 m through a vertical guide tube onto the disk. Following impact, animals remained under anesthesia on a heating pad (36.5–37.5 °C) until blood sampling and euthanasia. Sham animals underwent the identical anesthetic protocol, scalp incision, and periosteal elevation without weight impact; blood was collected immediately thereafter (time 0 h), without a post-procedural recovery period.

After the procedure, rats were transferred to a warmed recovery cage and observed continuously until spontaneous breathing normalized and the righting reflex returned. Injured animals regained the righting reflex within 3–8 min after impact, whereas sham animals recovered within 2–3 min after discontinuation of isoflurane. No animals exhibited skull fractures, prolonged apnea incompatible with survival, or extracranial complications during the hyperacute phase.

All TBI rats were allowed to recover fully from anesthesia and were returned to their home cages until sacrifice at the assigned time point. No unexpected behavioral abnormalities were observed prior to euthanasia. During the 72-h observation period, animals were monitored at least twice daily for general activity, grooming, posture, spontaneous locomotion, and feeding/drinking behavior. None of the animals developed seizures, decerebrate posturing, or severe motor deficits. Rats in the TBI groups displayed only transient hypoactivity and reduced exploratory behavior during the first 24 h, consistent with a severe diffuse injury phenotype.

### 2.3. Study Design and Sampling

Animals were randomly assigned to five groups (n = 10 per group): sham, 1 h, 6 h, 24 h, and 72 h post-injury. The study followed a cross-sectional, terminal sampling design in which each time point consisted of an independent cohort, and no longitudinal or repeated blood sampling was performed. Each rat underwent a single cardiac puncture immediately prior to euthanasia. Sham animals received the same anesthesia and surgical preparation without weight impact and were sampled at 0 h, serving as baseline controls for all groups. This design minimized physiological stress, avoided confounding effects of repeated phlebotomy, and ensured adherence to ethical standards for severe diffuse TBI models. Serum was separated by centrifugation at 3000 rpm for 10 min at 4 °C and stored at −80 °C until biochemical analysis.

### 2.4. Biochemical Analysis

Assays were performed using rat-specific ELISA kits for TNF-α (Rat Tumor Necrosis Factor Alpha ELISA kit; Cat. No. E0764Ra), IL-6 (Rat Interleukin 6 ELISA kit; Cat. No. E0135Ra), GFAP (Rat Glial Fibrillary Acidic Protein ELISA kit; Cat. No. E0538Ra), and insulin (Rat Insulin ELISA kit; Cat. No. E0707Ra), all supplied by Bioassay Technology Laboratory (Shanghai, China). These were standard sandwich ELISA kits designed for quantitative measurement of the respective analytes in rat serum.

In each assay, plates pre-coated with capture antibodies bound the target analyte from serum samples, followed by incubation with biotinylated detection antibodies and streptavidin–HRP. After washing to remove unbound reagents, substrate solution was added, generating a colorimetric signal proportional to analyte concentration. The reaction was terminated with stop solution, and absorbance was measured at 450 nm using a microplate reader. All samples were run in duplicate to ensure analytical reliability.

### 2.5. Statistical Analysis

All animals were sampled once only, and each biomarker measurement represents an independent subject at a defined post-injury time point. Data distribution was assessed using the Shapiro–Wilk normality test. Group comparisons were performed using one-way ANOVA followed by Tukey’s post hoc test for normally distributed variables and the Kruskal–Wallis test with Dunn’s correction for non-parametric data. Results are presented as mean ± standard deviation (SD). Statistical significance was defined as *p* < 0.05. Analyses were conducted using SPSS version 25.0 (IBM Corp., Armonk, NY, USA).

## 3. Results

**Figure 1:**
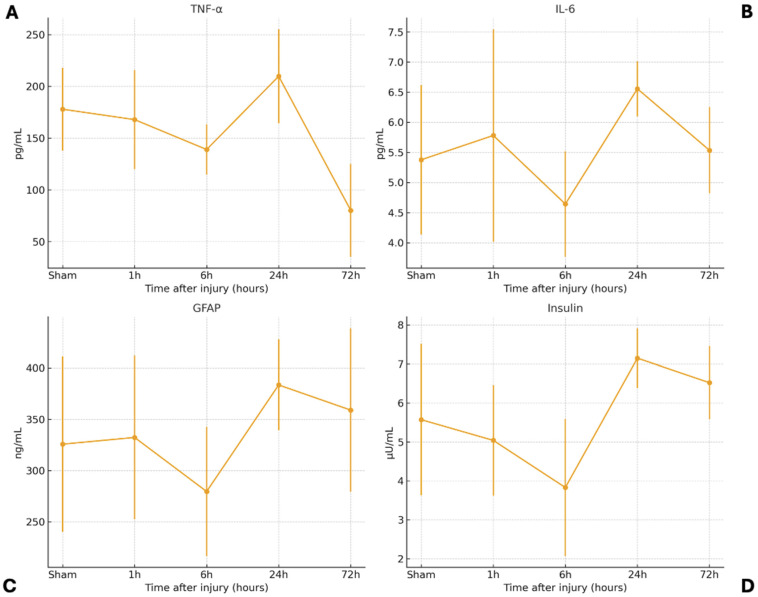
Temporal profiles of serum TNF-α (**A**), IL-6 (**B**), GFAP (**C**), and Insulin (**D**) levels following severe diffuse TBI in rats. Error bars represent ± SD; *p* < 0.05 vs. sham. Mean ± SD values for each time point are given in Table 1 (GFAP: Glial fibrillary acidic protein; IL-6: Interleukin-6; TNF-α: Tumor necrosis factor-alpha).

The temporal serum profiles of TNF-α, IL-6, GFAP, and insulin following diffuse TBI are summarized in Table 1. Each biomarker exhibited a distinct kinetic pattern that corresponded to different phases of the secondary injury cascade (Figure 1).

### 3.1. Serum TNF-α Levels

Serum TNF-α levels showed a mild decrease from the 1 h group (171.9 ± 50.3 pg/mL) to the 6-h (139.0 ± 24.2 pg/mL) relative to sham group (177.9 ± 39.9 pg/mL). Then, a significant increase was observed at 24 h (209.8 ± 45.4 pg/mL; *p* < 0.05) before declining sharply at 72 h (80.2 ± 44.8 pg/mL; *p* < 0.05) (Figure 1A). ANOVA demonstrated significant differences among groups (F = 11.54, *p* < 0.001). Tukey post hoc analysis confirmed that 24 h levels were higher than those at 6 h (*p* = 0.026) and 72 h (*p* < 0.001), while 72 h values were significantly lower than sham and 1-h (*p* < 0.001 and *p* = 0.0007, respectively). The mean serum TNF-α concentrations demonstrated a biphasic pattern over time indicating an early inflammatory response peaking at 24 h and subsiding thereafter, consistent with a classical acute phase pattern.

### 3.2. Serum IL-6 Levels

At 1 h, IL-6 levels remained stable (5.38 ± 1.24 pg/mL), followed by a mild decline at 6 h (4.64 ± 0.88 pg/mL). A significant secondary rise was observed at 24 h (6.56 ± 0.46 pg/mL; F = 2.93, *p* = 0.029), which remained slightly elevated at 72 h (5.53 ± 0.71 pg/mL) (Figure 1B). Tukey’s post hoc test indicated that IL-6 levels at 24 h were significantly higher than at 6 h (*p* = 0.0147). This kinetic profile reflects early suppression followed by reactivation of cytokine signaling during the subacute phase suggesting a secondary wave of cytokine activity associated with delayed glial modulation and repair.

### 3.3. Serum GFAP Levels

GFAP levels remained stable at 1 h (332.3 ± 79.8 pg/mL) and decreased slightly at 6 h (279.6 ± 62.9 pg/mL). A progressive increase was observed thereafter, peaking at 24 h (383.5 ± 44.2 pg/mL) and declining modestly by 72 h (359.0 ± 79.6 pg/mL) (Figure 1C). Although ANOVA did not show a statistically significant difference among groups (F = 1.91, *p* = 0.124), the temporal trend suggests delayed astroglial activation consistent with cytoskeletal disruption and reactive gliosis.

### 3.4. Serum Insulin Levels

Serum insulin levels decreased at 6 h (3.83 ± 1.76 μU/mL) compared with sham (5.57 ± 1.94 μU/mL) and 1 h (5.04 ± 1.42 μU/mL). A significant rise was subsequently observed at 24 h (7.15 ± 0.77 μU/mL) and 72 h (6.52 ± 0.94 μU/mL) (Figure 1D). ANOVA revealed significant group differences (F = 4.98, *p* = 0.0017). Tukey’s test showed higher insulin levels at 24 h (*p* = 0.0015) and 72 h (*p* = 0.0148) compared with 6 h. These findings indicate early metabolic suppression followed by recovery during the subacute phase.

**Figure 2:**
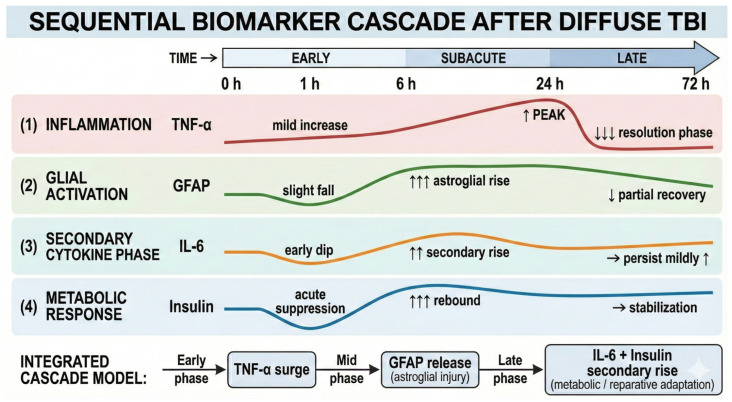
The sequence of inflammatory, glial, and metabolic responses following TBI. Primary mechanical impact is followed by an acute inflammatory surge (TNF-α), a subacute IL-6 rise, delayed astroglial activation (GFAP), and subsequent metabolic adaptation (insulin).

Collectively, the cytokine responses clearly preceded the rise in GFAP and insulin, delineating a coherent temporal cascade that transitions from acute inflammation to astroglial activation and subsequent metabolic regulation. The temporal trajectories of each biomarker are illustrated in Figure 1, demonstrating distinct kinetic profiles across inflammatory cytokines, astroglial markers, and metabolic hormones. A complementary overview of this integrated cytokine–glial–metabolic sequence is provided in Figure 2, which summarizes the ordered progression of secondary injury mechanisms following diffuse TBI.

## 4. Discussion

Traumatic brain injury triggers a complex sequence of neuroinflammatory, glial, and metabolic processes that evolve over time and greatly influence neurological outcomes [2,3,7]. Experimental diffuse TBI models, such as the Marmarou weight-drop paradigm, replicate acceleration–deceleration forces characteristic of human closed-head injuries and allow systematic evaluation of secondary injury mechanisms across defined time points [16,17]. Time-resolved biomarker profiling provides important insights into the dynamic nature of neuroinflammation and astroglial responses, offering greater translational relevance than single-time-point measurements. As shown in our previous work using the same model, longitudinal sampling enables detailed mapping of biomarker trajectories and supports improved interpretation of secondary injury progression [18].

Incorporating temporal biomarker kinetics strengthens the mechanistic understanding of secondary injury and supports the development of dynamic, phase-specific therapeutic strategies. In this study, each biomarker exhibited a characteristic temporal pattern that reflects the multifaceted progression of secondary injury. TNF-α and IL-6 highlighted the early inflammatory phase, GFAP indicated delayed astroglial disruption, and insulin reflected subsequent metabolic adaptation. Collectively, these trajectories illustrate the sequential activation of inflammatory, glial, and endocrine pathways following diffuse TBI. Although the biomarkers studied are not mechanistically novel, their synchronized temporal mapping provides a framework for selecting optimal sampling windows for transcriptomic, histological, or proteomic assays in future mechanistic studies.

### 4.1. Inflammatory Cytokine Response: TNF-α and IL-6 as Early Pro-Inflammatory Markers

TNF-α is predominantly released by activated microglia and astrocytes during the acute phase of injury, where it contributes to secondary neuronal damage by promoting apoptotic signaling, disrupting the BBB, and facilitating leukocyte recruitment [2,19]. Through TNF receptor 1-dependent pathways, TNF-α directly induces neuronal apoptosis [6,7]. The present findings further support the role of TNF-α as an early, short-lived, yet potent mediator of the acute inflammatory response, consistent with prior studies reporting peak levels within 6–24 h after injury [6,8]. The marked decline by 72 h likely reflects the activation of anti-inflammatory feedback mechanisms, potentially involving IL-10 signaling and nuclear factor (NF-κB) downregulation [20].

IL-6 exhibited a comparable yet temporally delayed pattern, characterized by a modest reduction at 6 h followed by a pronounced secondary rise at 24 h. This biphasic response underscores its dual function in early pro-inflammatory amplification and later regenerative signaling [21]. Beyond its inflammatory role, IL-6 promotes neuroprotection by supporting astroglial proliferation, neurotrophic factor release, and synaptic remodeling through janus kinase/signal transducers and activators of transcription (JAK/STAT) and cAMP-response element binding protein (CREB)-mediated pathways [22,23]. As highlighted by Gan et al. (2019), IL-6 and TNF-α together form a coordinated cytokine network that links neuroinflammation with neuronal survival after TBI [22]. The delayed elevation of IL-6 observed here aligns with reports indicating that cytokine reactivation coincides with glial scar formation and early reparative gliosis rather than sustained inflammation [20,24]. Continued IL-6 elevation at 72 h may reflect ongoing astroglial remodeling, consistent with chronic-phase cytokine trajectories documented in translational studies by Thelin et al. (2019) and Newcombe et al. (2022) [9,10].

Finally, ferritin biology provides additional mechanistic insight into the inflammatory cytokine responses observed here. Panther et al. (2022) describe ferritin as both protective—through iron sequestration—and detrimental when clearance pathways are overwhelmed, leading to iron-driven oxidative stress [25]. Ferritin-related processes highly relevant to our model include microglial ferritin uptake and degradation via autophagy or proteasome pathways, the release of redox-active iron that can amplify TNF-α and IL-6 production, modulation of astrocyte- and microglia-driven responses, and potentiation of reactive oxygen species (ROS)-mediated cellular injury. Although ferritin was not quantified in the present study, the temporal cytokine patterns identified—particularly the secondary IL-6 surge and the early TNF-α elevation—are compatible with iron-associated inflammatory amplification described in ferritin-mediated neurotoxic pathways.

### 4.2. Astroglial Response: GFAP Reflects Structural Injury

GFAP, an intermediate filament protein expressed exclusively by astrocytes, is widely recognized as one of the most robust biomarkers of glial injury, reflecting both astrocytic structural compromise and BBB permeability changes [24]. Circulating GFAP levels correlate strongly with the magnitude of astroglial damage and are considered reliable indicators of secondary injury progression rather than immediate mechanical disruption [26,27]. Consistent with this, authors highlighted the superior sensitivity of serum GFAP for detecting astrogliosis and glial cytoskeletal disintegration in TBI [12]. Recent clinical investigations further demonstrate that GFAP correlates robustly with injury severity and outperforms other glial markers such as S100 calcium binding protein B (S100B) and ubiquitin carboxyl-terminal hydrolase L1 (UCH-L1) in predicting computed tomography-positive lesions [26]. Longitudinal studies by Newcombe et al. (2022) revealed that GFAP may re-emerge years after trauma, implying persistent astroglial activation in delayed neurodegeneration [10]. Complementary evidence from McGinn and Povlishock (2016) indicates that GFAP release predominantly reflects astrocytic injury secondary to diffuse axonal injury and BBB breakdown, rather than direct neuronal necrosis [2].

In the present study, GFAP demonstrated a characteristically delayed kinetic profile, with concentrations increasing most prominently between 6 and 24 h after injury. This temporal lag is consistent with GFAP’s biological role as a marker of cytoskeletal disruption and reactive astrogliosis rather than acute necrotic release. Prior experimental work shows that serum GFAP typically peaks within 24–48 h following TBI, coinciding with astroglial necrosis and the early development of reactive gliosis—findings that closely parallel the temporal pattern observed here [28,29]. Although intergroup differences did not reach statistical significance, a clear upward trajectory was evident, with peak levels at 24 h and partial decline by 72 h. This trend supports the concept that astroglial activation and cytoskeletal remodeling occur in a delayed but progressive manner relative to the initial cytokine surge [8,30].

Emerging neuropharmacological evidence offers an additional mechanistic perspective: the sigma-1 receptor has been identified as a key regulator of endoplasmic reticulum stress, mitochondrial stability, calcium homeostasis, and reactive gliosis. In this context, the early dip and later rise in GFAP—as well as the biphasic cytokine pattern—may partially reflect sigma-1-linked glial and microglial responses [31]. Although sigma-1 receptor activity was not directly measured, our temporal dataset provides a conceptual framework for future studies evaluating sigma-1 receptor ligands as modulators of astroglial reactivity in early secondary injury.

Taken together, the gradual rise in GFAP observed in our study mirrors established subacute astroglial dynamics documented in both preclinical and clinical literature. This pattern likely represents the transition from initial neuroinflammation to reactive gliosis and may reflect coordinated microglial–astroglial interactions during glial scar formation, in line with diffuse axonal injury pathophysiology [19,24,32].

### 4.3. Metabolic Adaptation: Insulin Regulates Energy Homeostasis

Experimental and clinical evidence demonstrates that insulin signaling is tightly integrated with cerebral glucose utilization and mitochondrial function and is highly vulnerable to traumatic injury [14,15]. Early after TBI, acute catecholamine surge and systemic stress commonly induce transient hypoinsulinemia, whereas later phases are frequently characterized by compensatory hyperinsulinemia as part of metabolic realignment and endocrine recovery [19].

In the present study, serum insulin exhibited a clear biphasic pattern, with an early decline at 6 h followed by a robust rebound at 24 and 72 h. The initial reduction likely reflects transient suppression of hypothalamic–pituitary regulation and acute neuroendocrine stress, whereas the subsequent elevation suggests activation of counterregulatory hormonal pathways and restoration of metabolic homeostasis. Similar temporal dynamics have been reported by Pearn et al. (2017) and Duman et al. (2008), who described early hypoinsulinemia followed by delayed hyperinsulinemic compensation in experimental TBI models [12,19].

The rebound observed between 24 and 72 h may also reflect insulin-mediated neuroprotective processes. Both insulin and IGF-1 enhance neuronal survival by facilitating glucose uptake and activating pro-survival signaling cascades such as protein kinase B (Akt) and 5′ adenosine monophosphate-activated protein kinase (AMPK), thereby reducing oxidative stress and supporting mitochondrial integrity [13,14]. These mechanisms echo the biphasic endocrine responses described in both preclinical and clinical studies, reinforcing the concept that metabolic adaptation evolves in parallel with inflammatory and glial responses.

Importantly, dysregulated insulin signaling—manifesting as insulin resistance or impaired glucose metabolism—is frequently documented after TBI and has been linked to worse neurobehavioral outcomes and prolonged neuronal vulnerability [6,7]. The partial normalization of insulin levels observed during the subacute phase in this study therefore may signify the gradual restoration of cerebral energy utilization and attenuation of systemic stress, contributing to improved neuronal metabolic stability.

### 4.4. Integrative Pathophysiology of TBI: Sequential Cytokine–Glial–Metabolic Cascade

Taken together, the temporal trajectories of TNF-α, IL-6, GFAP, and insulin demonstrate a tightly coordinated interaction among neuroinflammatory, glial, and metabolic pathways. These findings outline a hierarchical sequence in which (1) an early surge in TNF-α reflects rapid pro-inflammatory activation, (2) subsequent GFAP elevation marks delayed astroglial structural disruption, and (3) later rises in IL-6 and insulin indicate secondary cytokine modulation and metabolic adaptation. This progression aligns with frameworks describing a transition from acute cellular injury to subacute reparative responses [1,19].

This temporal hierarchy aligns closely with the triphasic framework proposed by Najem et al. (2018) and Thelin et al. (2019), encompassing acute neuroinflammatory activation, intermediate glial reorganization, and later metabolic remodeling [1,9]. Moreover, the biphasic cytokine profile observed here mirrors human biomarker kinetics reported by Newcombe et al. (2022), who demonstrated early TNF-α/IL-6 elevations followed by delayed GFAP reactivation, suggesting prolonged astroglial involvement in post-traumatic neurodegeneration [10]. These findings further support the translational concept advanced by Wang et al. (2018) and Doust et al. (2026), emphasizing that multimodal biomarker panels improve diagnostic and prognostic performance beyond the utility of single markers [32,33].

Lucke-Wold et al. (2025) further emphasize that key drivers of secondary injury—oxidative stress, mitochondrial dysfunction, neuroinflammation, and endoplasmic reticulum stress—can be modulated by targeted supplement and nutraceutical interventions [34]. Their work underscores that biomarkers such as TNF-α, IL-6, and glial proteins evolve dynamically over time, shaping therapeutic windows and treatment responsiveness. These concepts closely align with our temporal dataset and support the relevance of serial cytokine–glial–metabolic profiling for identifying optimal intervention timing after TBI.

Growing evidence from exosomal and proteomic studies reinforces this multidimensional approach, with glial (GFAP, S100B), axonal (Neurofilament light chain, UCH-L1), and metabolic (insulin, insulin like growth factor-1) proteins collectively outperforming individual biomarkers in predicting injury severity and outcome [30,35]. In this context, the concurrent assessment of TNF-α, IL-6, and GFAP provides complementary insight into inflammatory and astroglial processes, whereas serum insulin offers an additional index of systemic stress and metabolic recovery. Clinically, such dynamic profiling could facilitate early injury stratification, optimize therapeutic timing, and improve monitoring of recovery trajectories.

Given the expanding recognition of GFAP and IL-6 as biomarkers of BBB integrity and glial activation, their integration alongside neuroimaging and functional scoring may refine prognostic frameworks in moderate-to-severe TBI. Future investigations should incorporate histopathological validation, brain tissue cytokine quantification, and neurobehavioral correlates to map mechanistic links between biomarker kinetics and structural injury. Additional molecules—including IL-1β, S100B, and neuron-specific enolase—may further enhance predictive accuracy within emerging multimarker platforms [1,19]. Recent proteomic and exosomal studies also underscore the utility of multiplex panels combining glial, axonal, and metabolic biomarkers to achieve superior precision in prognostication and treatment guidance [33,35].

Finally, the present findings highlight the necessity of serial rather than single-time-point biomarker sampling to capture the dynamic evolution of secondary injury. This principle echoes Neher et al. (2014), who emphasized that ideal biomarkers must reflect the temporal progression of tissue injury and repair [24].

Several limitations warrant consideration. The exclusive use of serum measurements without parallel histopathology restricts mechanistic interpretation; thus, the present results should be viewed as descriptive temporal patterns rather than evidence of causal pathways. Inclusion of only male rats prevents assessment of sex-dependent differences in cytokine or metabolic responses. Additionally, while the sample size was adequate for biochemical comparisons, larger cohorts may be necessary to detect subtle temporal effects. Future work should extend these findings by integrating tissue-level analyses, neurobehavioral outcomes, and molecular pathway assessment—including IL-10–mediated neuroprotection, nuclear factor-κB regulation, and insulin–AMPK signaling—to further clarify the interplay among inflammatory, glial, and metabolic networks following TBI [20].

## 5. Conclusions

This experimental study characterizes the temporal dynamics of serum TNF-α, IL-6, GFAP, and insulin following diffuse TBI, revealing a coordinated sequence of inflammatory, glial, and metabolic responses. TNF-α exhibited an early peak at 24 h, consistent with acute neuroinflammation; GFAP increased subacutely, reflecting delayed astroglial activation; and both IL-6 and insulin demonstrated secondary rises, indicating engagement of reparative cytokine signaling and metabolic recovery pathways. Together, these findings underscore the interdependence of immune, glial, and endocrine mechanisms in shaping the evolution of secondary brain injury.

The results highlight the value of serial biomarker profiling for defining injury phases and suggest that multimodal panels incorporating cytokine, glial, and metabolic markers may improve characterization of TBI pathophysiology. In particular, GFAP and IL-6 emerge as promising subacute indicators of astroglial injury and systemic response, with potential relevance for translational prognostication. While not mechanistic, these integrated biomarker trajectories highlight distinct and sequential phases of inflammatory activation, astroglial response, and metabolic adjustment. Such temporal mapping is methodologically valuable for experimental design in future mechanistic TBI studies. Further work combining serum biomarkers with histological, molecular, and functional assessments will be required to define causal pathways and strengthen translational applicability.

## Figures and Tables

**Table 1:** Serum TNF-α, IL-6, GFAP, and insulin levels (mean ± SD) at each time point following diffuse TBI in rats.

Time Point Following Diffuse TBI	TNF-α	IL-6	GFAP	Insulin
	(pg/mL)	(pg/mL)	(pg/mL)	(µU/mL)
	Mean ± SD	Mean ± SD	Mean ± SD	Mean ± SD
**Sham**	177.9 ± 39.9	5.38 ± 1.24	325.7 ± 85.3	5.57 ± 1.94
**1st h**	171.9 ± 50.3	5.78 ± 1.76	332.3 ± 79.8	5.04 ± 1.42
**6th h**	139.0 ± 24.2	4.64 ± 0.88	279.6 ± 62.9	3.83 ± 1.76
**24th h**	209.8 ± 45.4 *	6.56 ± 0.46 *	383.5 ± 44.2	7.15 ± 0.77 *
**72nd h**	80.2 ± 44.8 *	5.53 ± 0.71	359.0 ± 79.6	6.52 ± 0.94 *

* *p* < 0.05 vs. 6 h (TNF-α, IL-6, Insulin); TNF-α at 72 h also significantly lower vs. sham and 1 h (*p* < 0.001 and *p* = 0.0007, respectively) (GFAP: Glial fibrillary acidic protein; IL-6: Interleukin-6; TBI: Traumatic brain injury; TNF-α: Tumor necrosis factor-alpha).

## Data Availability

All data generated or analyzed during this study are included in this article. Additional information is available from the corresponding author upon reasonable request.

## Abbreviations

The following abbreviations are used in this manuscript:

BBB	Blood–brain barrier
GFAP	Glial fibrillary acidic protein
IL-6	Interleukin-6
TBI	Traumatic brain injury
TNF-α	Tumor necrosis factor-alpha
